# Exploring the Opinions of Irish Beef Farmers Regarding Dairy Beef Integration

**DOI:** 10.3389/fvets.2021.660061

**Published:** 2021-06-14

**Authors:** James W. Maher, AnneMarie Clarke, Andrew William Byrne, Rob Doyle, Martin Blake, Andrew Cromie, Damien Barrett

**Affiliations:** ^1^Department of Agriculture, Food and the Marine, Dublin, Ireland; ^2^Irish Cattle Breeding Federation, Cork, Ireland

**Keywords:** dairy beef integration, beef farmers, male calves, qualitative research, Ireland

## Abstract

**Background:** There has been very little previous research in Ireland on the opinions of farmers regarding dairy beef integration. The need for increased dairy beef integration has assumed a greater importance in Ireland in recent years due to a rapid expansion in dairy production, and associated increase in numbers of male dairy calves born on Irish farms. The objective of this study was to explore beef farmers' views on a broad range of issues related to dairy beef integration, using a survey methodology. The survey was distributed to approximately 4,250 beef farmers via email and 1,203 participated in the study.

**Results:** The sample was composed almost entirely of beef farmers, although a very small proportion also had a dairy enterprise on their farm. Eighty percent of the farmers were concerned with the increase in the number of male dairy calves in recent years. Fifty seven percent of farmers responded that they were not willing to rear dairy bred calves for beef. Limousin, Aberdeen Angus and Hereford were the breeds farmers would be most willing to rear for beef. Good health, breed, and conformation were ranked as the main factors calf rearers consider when buying calves. Expectation of poor profit margin, expectation of poor-quality calves, and price volatility/market uncertainty were the top ranked factors dissuading farmers from rearing dairy calves for beef. The main themes arising from the qualitative question related to beef price/ability to make a profit, breed, and calf quality.

**Conclusions:** While it is concerning that the majority of respondents expressed an unwillingness to rear dairy bred calves for beef, approximately a quarter of beef farmers indicated a willingness to rear beef-sired dairy calves for beef. In the qualitative responses, farmers described how their concerns about calf quality and their ability to make a profit from dairy bred calves would make it difficult for them to rear these calves for beef. Future strategy will have to consider how these challenges can be overcome and the issues of who bears the risks and costs associated with greater integration will have to be carefully considered.

## Introduction

In Europe, dairy herd numbers were largely static from the mid 1980s until 2015 (1). Following the abolition of European milk quotas in 2015, there was a substantial expansion in the Irish dairy industry, with the national dairy herd increasing by 27% between 2013 and 2018 (2). A consequence of the increase in dairy cows is the increased number of male dairy calves. Renaud et al. (3) described how the health and welfare of male dairy calves have been lingering issues in dairy sectors around the world. While over 60% of the beef produced in Ireland comes from the dairy herd (4), greater integration between the dairy and beef sectors will be required in the coming years in order to sustainably manage the increasing number of male dairy calves.

In New Zealand and Australia, the majority of male dairy calves are transported long distances to be slaughtered shortly after birth (5). Much of the Irish dairy industry has modelled itself on New Zealand's pasture-based production system (6). In Europe and North America, the majority of male dairy calves contribute to the red meat industry, but the move to dairy specific type genetics (e.g., jersey breed) in Ireland has led to increased numbers of calves with inferior beef characteristics, which beef farmers have difficulty in making a reasonable economic margin on. In Europe, the issue of managing these male dairy calves has become prominent in recent years, attracting significant media attention in Ireland (7) and the UK (8).

Unlike most other EU member states, in Ireland milk production is mainly pasture based with seasonal spring calving (9, 10). As such, there is a seasonal peak in the number of male dairy calves born on Irish dairy farms each spring. A significant outlet for these calves has been through export to continental Europe for veal production (11). While Irish farm organisations are anxious to maintain these export markets, the issue of live exports has been contentious at EU level, with an EU parliamentary committee currently examining the welfare of unweaned animals during transport over long journeys. Live animal exports are also an issue of concern to non-governmental animal welfare organisations. Therefore, like several other countries, issues around breeding male dairy calves and their role remain contentious in Ireland (12). The development of local veal or dairy beef production have been identified as alternative approaches to the movement of dairy calves in Canada (13).

The current study is necessary because it is very important to assess the opinions of the stakeholders of new policies which are likely to affect them. For example, previous Irish research on welfare schemes for suckler farmers demonstrated how including farmers in the early stages of designing new initiatives helps ensure that schemes are practical and relevant (14). Very little previous work has examined Irish beef farmers' views on dairy beef integration and this study aims to fill a lacuna in the research literature. The specific objective of this study was to gather information on the attitudes of Irish beef farmers regarding the large number of male dairy calves, assess their willingness to rear these calves for beef, and get their thoughts on a broad range of factors related to greater integration including breed, practical barriers, and preferred models of integration.

## Methods

### Survey Design

A self-completion survey using the *SurveyMonkey* software package was created by the One Health Scientific Support (OHSS) team within the Irish Department of Agriculture, Food and the Marine (DAFM), with expertise in social science, veterinary, epidemiology and animal science backgrounds. Input was also provided by senior official veterinarians within DAFM and by colleagues in the Irish Cattle Breeding Federation (ICBF). In total, there were seventeen questions to be answered. An information section was presented at the outset, assuring potential participants that their anonymity would be protected and that their participation was voluntary, and the first question then asked respondents if they consented to participate in the study. Fifteen quantitative questions, comprising multiple choice and ranking style questions, were included in the survey. Where multiple responses were available the order of the options was randomised to minimise responder bias. In the ranking style questions, the participants had to rank each of the options; with 1 being what they felt the most important factor was. The method used for scoring these responses was to assign a reverse score, that is, the most important factor, ranked by a farmer at number 1, was given a score of 7. The weighted average was then used to determine the rankings. It was made compulsory to provide a response to each question appropriately before one could progress to answering the next question. Section breaks were also used, in conjunction with automatic skipping, to bring respondents to the end of the survey when they had completed all questions of relevance to their demographic cohort.

The final question was qualitative in style, using a free text field, where participants were asked to provide any suggestions that they had regarding how dairy beef animals could be better integrated into beef rearing/finishing systems. Qualitative research asks participants “to describe their experiences in ways that are meaningful to them” (15). Although the findings of such research cannot be generalised to other contexts, this method helps provide a greater understanding of certain issues, through the unique perspective of the participants. The rationale for incorporating a qualitative component to this survey was to help improve DAFM's understanding of beef farmers' opinions on topics related to dairy beef integration. A list of the questions asked, and available responses, is available in the Supplementary Table 1.

### Data Collection

Beef production is the most common farm type in Ireland, with over 70,000 farms (16). The OHSS team issued a link to the survey using email to a sample of approximately 4,250 predominately beef farmers, through a mailing list held by the ICBF on June 15, 2020. These 4,250 farmers have subscribed to ICBFs HerdPlus service (17) which provides them with on-farm performance data that can be used to help increase farm profit. One email reminder was subsequently issued and, by the closing date of June 28, 2020, a total of 1,227 responses had been received. It is not possible to determine an accurate response rate given the possibility that recipients of the email containing the survey link may have forwarded it to others to complete. A total of 1,203 respondents (98%) consented to participate in the study. The authors acknowledge the possibility of selection bias that may arrive from self-selection to participate in the study.

### Data Analysis

SurveyMonkey aided the presentation of the results of the quantitative questions (Q2–Q16), generating tables and graphs presenting the results of each question; the output file is available in the Supplementary Data Sheet. SurveyMonkey was used to generate rating scales for ranking style questions, as in previous research including Sayers et al. (18). Regarding the qualitative question, a total of 541 responses were received (representing 45% of the respondents who had consented to participate in the study). The first step in the analysis of these responses was to identify any unusable replies, for example, where the respondent had indicated that they had no further comment. At this stage, 32 responses were removed, leaving 509 substantive responses for further analysis, amounting to a total word count of almost 15,000 words.

The next step was to read through each of the individual responses and assign a tag, or multiple tags where appropriate, categorising the responses as falling under specific themes. There is evidently a strong subjective element to any such categorisation exercise as judgment calls frequently have to be made in deciding what theme most accurately represents the content of the response (19). It is not claimed that the list of themes used to categorise the qualitative responses is exhaustive, rather the themes cover those topics which could be clearly defined and which arose in multiple responses. One hundred and twenty three of the responses referred, in whole or partially, to issues that could not be categorised clearly within these chosen themes. In this paper only the most common themes will be presented and discussed. Select, representative quotes will be used to demonstrate the attitudes of the beef farmers. Quotes were chosen based on whether the quote is “illustrative of the point the writer is making about the data, it is reasonably succinct, and it is representative of the patterns in data” (20).

## Quantitative Results

### Completion

In all, 902 respondents (75% of those who consented to participate) completed all fifteen quantitative questions.

### Demographics (Q2–Q5)

In respect of farm type, participants were permitted to select all farm enterprise types that they currently engaged in. Suckler to weanling (58%) was the most common enterprise type. This enterprise involves beef cows producing a calf and rearing that calf until it is weaned off milk at approximately 9 months of age. The progeny is then sold. The suckler to beef farming was the second most common enterprise (35%). The difference in this enterprise to the previous is that the progeny are not sold as weanlings but reared on farm until fit for slaughter. Regarding the “other” option, in the free text field, farmers frequently mentioned pedigree beef (e.g., specific breeds such as Angus) and sheep. Sixty five percent of the respondents were aged over 45 years. The geographic breakdown by province was: Munster−35%, Leinster−25%, Ulster−8%, Connacht−32%. Regarding work pattern, 64% of participants identified as being part-time farmers, with 36% describing themselves as farming on a full-time basis.

### Herd Statistics (Q6)

The largest group of farmers had between 51 and 100 cattle on their farm (36%), with a further 31% of farmers having between 21 and 50 cattle. A small proportion of participants had fewer than 20 (9%) or more than 200 cattle (6%).

### Breed (Q7)

Farmers were asked to select which breeds (including crossbreed varieties) they would be willing to rear or finish for dairy bred beef production. Participants were permitted to select multiple options. Limousin (59%), Aberdeen Angus (51%) and Hereford (45%) were the three most popular breeds. Regarding the “other” option (19%), the most popular breeds mentioned in the free text field were Charolais, Simmental, and Aubrac.

### Attitudes to Dairy Beef Integration (Q8–Q11)

#### Number of Male Dairy Calves

Eighty percent of participants stated that they were concerned about the increased number of male dairy calves in recent years. An interesting observation regarding this question was that 121 participants (10% of those who had consented to take part) dropped out of the survey at this juncture, without providing a response.

#### Factors Considered When Buying Calves

Respondents were asked to rank the factors they considered most important when buying calves. Farmers felt good health (5.45), breed (5.08), and conformation (4.85) were the three main factors calf rearers look for when buying calves. Conformation (4.26), good health (3.88), and breed (3.6) were the three highest ranked factors beef finishers would consider when buying dairy bred beef animals.

#### Willingness to Rear Calves From Dairy Farms

Participants were able to select multiple options, that is, participants could say they currently rear beef-sired dairy calves and they would also be willing to rear dairy-sired dairy calves. Sixteen per cent of respondents already rear beef-sired dairy calves and 14% already rear dairy-sired dairy calves for beef. Fifty-seven per cent of farmers indicated that they were not willing to rear dairy bred calves for beef. Twenty-four per cent of farmers expressed a willingness to rear beef-sired dairy calves. Just seven per cent of farmers were willing to rear dairy-sired dairy calves.

### Genomic Verification (Q12)

Approximately half of respondents felt that genomic verification was either an extremely important (22%) or very important (26%) consideration. Only 9% felt that it was not important at all.

### Barriers to Greater Dairy Beef Integration (Q13–Q14)

Expectation of poor profit margin (5.39), expectation of poor-quality calves (5.34), and price volatility/market uncertainty (5.1) were the three top ranked factors dissuading farmers from rearing dairy calves for beef. It is worth noting that 120 participants (10% of those who had consented to take part) dropped out of the survey at this point, without providing a response to this question. While poor profit margin on investment was the most significant barrier, it is worth noting that farmers did not see a lack of husbandry skills to care for dairy calves as being an impediment to rearing these animals.

### Future Dairy Beef Integration (Q15–Q16)

#### Strategy

When asked to rank potential strategies in order of how effective farmers thought these strategies would be in improving dairy beef integration, guaranteed pricing mechanisms (6.12) were the most popular factor by a considerable margin. Grants for improving dairy beef infrastructure (4.61) and a system to source quality calves (4.47) were ranked as the second and third most effective strategies, respectively.

#### Models of Integration

Given the complexity of the models of dairy beef integration being presented as options, detailed descriptions of these options were provided for the farmers' information. The most popular model ranked was a system where a beef farmer rears and/or finishes dairy bred calves with guaranteed pricing mechanism, with the beef farmer having ownership of the calves (4.75). The second and third most popular options were a birth to slaughter production system (4.6) and contract rearing for dairy farmers (4.41). A birth to slaughter production system was described as a fully integrated system where there are contracts between the dairy farmer to supply the beef farmer with an agreed type of calf, and a contract with the beef processor to supply minimum agreed price (or bonus system) to the beef farmer who rears this animal to the agreed specification. Contract rearing refers to the practice whereby calves are moved to another farm for a different farmer to rear them in return for an agreed payment. The dairy farmers retain ownership and by using contract rearing they can free up labour, accommodation facilities, and grazing land on their own farm, allowing them to focus on their dairy enterprise activities. The least preferred option was to have no change to the status.

## Qualitative Results

### Price/Profit (*n* = 140)

The most common category of responses referred to the price paid to the beef farmer by the factory or farmers being able to make a profit when they sell their animals to the factory. Many respondents spoke about not receiving a fair price that was reflective of the huge amount of time and labour they have put into rearing their animals for beef, resulting in a very poor profit margin or, in some cases, farmers making a loss. Fifty-four participants (10% of those who provided a free text response) mentioned the need for guaranteed pricing mechanisms, with most referring to how such systems would lead to greater certainty that farmers can make a living.

The interaction between ensuring a fair price and calf quality arose frequently, as described by this farmer: “*need for certainty regarding sourcing of good quality calves, at a fair price, and subsequent certainty regarding margins to the beef farmer will be crucial if this system is going to stand the test of time*.” Notably this several respondents under this theme recognised that while price is important, other factors, particularly the health of the animals, are also very important; “*Price is a factor but knowing we can get an animal that is well bred, healthy and has a chance at finishing at 21 months is crucial to us.”*

While there was generally little mention of specific organisations in the responses, several respondents under this theme spoke about price fixing being a long standing issue and the need for Government or independent regulators to intervene to prevent this practice occurring.

### Breed (*n* = 127)

Responses referring to breed could be broadly split into two themes. Firstly, respondents spoke about the need for dairy farmers to reduce the use of breeds with poor beef merit in their herds. Jersey/Jersey cross and Holstein were the most frequently mentioned in negative terms. The second sub-theme was the desire for greater use of dual-purpose or continental breeds in dairy herds so that beef farmers could rear the male dairy calves for beef more efficiently. The British Friesian breed were mentioned the most often in this regard. Other breeds included Fleckvieh and Montbéliarde. The use of Angus or Hereford sires for breeding dairy cows would also be beneficial for beef farmers.

Farmers spoke about how the disparity between beef and dairy breeds is a recent development, recalling greater use of dual purpose breeds by dairy farmers in the past; “*I am around long enough to have seen the finest of beef produced from British Friesian cows, the dairy industry has moved away from that type of animal, they're seen as inefficient*.” This idea of dairy farmers having to make a compromise on productivity for milk production in order to have calves that beef farmers would be willing to rear recurred throughout the survey responses.

### Calf Quality (*n* = 91)

The vast majority of these farmers referred to how the quality of dairy calves would need to improve significantly before they would consider rearing these animals for beef. Respondents described how it is already challenging to maintain a living with good quality beef animals without having to rear animals of lower beef merit; “*Trying to survive as a suckler farmer is hard with good cattle, l*et al*one diluting your herd with cattle that look like marathon runners.”* Several respondents under this theme spoke in disparaging terms about the poorer beef characteristics of calves coming from dairy farms, especially the difficulty in trying to fatten these animals, particularly for certain breeds such as Jersey-cross bred calves.

There was an overlap between responses mentioning calf quality and potential welfare issues, with several participants under this theme speaking about the need for urgent change to avoid serious welfare problems developing; “*If Ireland doesn't address the issue of calf quality soon there will be no one to rear these calves and we will end up … shooting calves”*. Farmers recognised that while changes in calf quality need to occur, this will mean that dairy farmers have to sacrifice some dairy productivity in order for beef farmers to be able to rear the male dairy calves for beef; “*Better quality calves from the dairy herd are needed, but striking the balance is impossible.”*

### Suckler Farming (*n* = 64)

The majority of responses under this theme mentioned how this farm enterprise type is already under severe pressure, with farmers describing the significant stress and financial hardship they are experiencing. This often coincided with a sense of frustration that suckler farmers are further challenged by having to deal with the unwanted by-products of the dairy sector; “*Suckler farmers just feel like we're being told we have to change what we do to accommodate the dairy sector, our years of breeding and work towards building quality beef herds is irrelevant.”*

Many farmers spoke about the need for greater financial assistance and how farmers engaging in this farm enterprise type required urgent protection. These comments often mentioned how they felt dairy farmers were being prioritised when they believe that beef farmers are in need of greater financial assistance. Several respondents under this theme spoke with a sense of pride about the quality of the product they have been producing, having engaged in careful planning and hard work over many years to improve their herds. These suckler farmers feel that they should be rewarded for the quality of beef they produce; “*suckler beef should be prime beef and marketed as a premium product*.” Following on from this many suckler farmers expressed worries that poorer quality beef coming from dairy herds would undermine their hard-earned reputations as quality beef producers.

### Grants/Subsidies (*n* = 60)

Under the European Union's Common Agricultural Policy, there are several subsidies which can be paid to farmers by government, to support farming practices that are beneficial to the sector. Respondents mentioned the need for financial support to help beef farmers with initial set up costs including the infrastructure for calf housing and calf feeding equipment. Several participants under this theme spoke about introducing financial supports to mitigate against the high costs of milk replacer due to having to rear these animals for longer, either by paying dairy farmers to rear the calves for longer before selling to the beef farmers or subsidising the cost of milk replacer if beef farmers are rearing the younger calves. The overlap between grants and beef prices was addressed by the following participant, who suggested that fair prices would reduce the need for grant aid; “*If there was profit in the business there would be no need for farmers to apply for grants*.”

### Anti-integration (*n* = 57)

These responses could be classified as farmers who are against the idea of integration of dairy and beef enterprise types. There was a strong sense of frustration that beef farmers are being required to solve the situation that has arisen with the large number of male dairy calves; “*yet again the beef farmer being asked to tidy up after greedy dairy men*.” This conflict also arose in the context of some farmers' opinions on the sustainability of the two enterprise types; “*Dairy calves are a by-product of intensive unsustainable dairy farming and should not be allowed to replace sustainable low input suckler beef farming*.”

### Awareness of Beef Merit in Dairy Bred Calves (*n* = 46)

Many farmers spoke about the loss of knowledge among dairy farmers about what constitutes a good animal for beef production; “*The vast majority of dairy farmers today wouldn't know a good beef animal if it kicked them as they are solely focused on milk production with little regard being given to the beef side and they no longer have the skills to recognise a decent animal with good conformation*.” The respondents felt that a singular focus on dairy traits and milk production has led to a large number of calves unsuitable for beef production.

## Discussion

To the authors' knowledge, this is the first survey to study Irish beef farmers' attitudes to the issue of dairy beef integration. The difficulty for beef farmers in making a profit on rearing dairy bred calves for beef, the lack of focus on beef characteristics in the breeding choices of dairy farmers, and the poor quality of calves coming from dairy herds were found to be the main challenges or perceived barriers to greater dairy beef integration.

### Price/Profit

The main themes arising from both the quantitative and qualitative parts of the survey was the apparent low prices received by farmers for their beef and, in particular, the poor profit margin they consider, that they make having reared their animals for beef production. Regarding factors dissuading farmers from rearing dairy calves for beef, an expectation of poor profit margin was ranked highest. Similarly, the number one ranked practical barrier to greater integration was expectation of a poor profit margin on investment. Furthermore, responses mentioning price or profit comprised the largest category emerging from analysis of the qualitative question. This echoes data on beef farming from Teagasc (21), an Irish state agency responsible for conducting agricultural research and providing agricultural advice to farmers, which described how “profitability at farm level is extremely low.” Recent media reports have highlighted the significant financial challenges facing Irish beef farmers, with the average beef farmer making a loss, before payments from various EU schemes are included (22). While beef profit margins are low (23), it is especially difficult to make a margin on poorer quality dairy bred animals that have poor conformation in comparison to other beef breeds. The issue of greater price certainty arose frequently in the qualitative responses and guaranteed pricing mechanisms were ranked as the number one strategy to improve dairy beef integration in the quantitative question. One farmer representative organisation, the Irish Cattle and Sheep Farmers Association, which represents beef and sheep farmers solely, has called for dairy farmers to pay beef farmers to rear their dairy bull calves, stating that “this problem cannot be resolved by transferring the risk to beef farmers who are already at breaking point” (24).

### Breed

Dairy bred Angus and Hereford calves were the breeds which beef farmers indicated they would be willing to rear for beef in both the quantitative and qualitative responses. Almost half of farmers indicated that genomic verification was either an extremely important or very important consideration, with several farmers also writing in the free text question about the need to be certain that the animals they purchase are as described. Negativity surrounding the use of Jersey or Jersey cross breed animals was a strong theme in the qualitative responses, as it was in our previous study of dairy farmers (25). However, this issue appears to attract disproportionate attention and it is difficult to reconcile the difference between the perception of how significant a problem these breeds represent and the fact that they represent a small, declining, proportion of the national herd (26). The sustained focus on the issue in the agricultural media (27) may help to explain why it arose so frequently in responses in this survey. This negative media coverage may also help explain the decline in Jersey usage in recent years. A recent initiative aimed at facilitating greater integration between Irish dairy and beef sectors, known as the ICBF's integrated dairy beef programme, is not using Jersey genetics (28).

### Barriers to Dairy Beef Integration

Eighty percent of beef farmers indicated that they were concerned by the increased number of male dairy calves in recent years. In our previous survey of dairy farmers (Maher et al., in press), when asked the same question, 58% of participants indicated that they were concerned about recent increase in the number of male dairy calves. It is understandable that a larger proportion of beef farmers are concerned by this development, given the strong sentiment expressed in the qualitative responses that beef farmers feel like they are being left responsible for rearing these male dairy calves, which they consider to be less efficient. The number of farmers who expressed anti-integration sentiments in the qualitative responses highlights the difficulty facing initiatives promoting greater dairy beef integration. While farm organisations are recognising that the percentage of beef being produced from dairy herds is likely to increase over the coming years (29), greater efforts need to be made to communicate to farmers about the potential benefits of integration for beef farmers and the broader agricultural sector in Ireland. A smaller number of beef farmers spoke about a range of other topics including genomic verification (*n* = 36), sexed semen (*n* = 23) and exports (*n* = 21); the latter two themes having been the main themes emerging from our previous survey of predominately dairy farmers (Maher et al., in press). This also suggests that the beef farmers are looking to other solutions that may solve the issue for surplus male dairy-bred calves rather than integration.

### Calf Quality/Beef Merit

While many of the respondents indicated in the free text response that the quality of beef from dairy herds was inferior, this has been disputed in the research literature (30). Concerns around the quality of calves coming from the dairy herd are likely driving the fact that 57% of farmers indicated they would not be willing to rear dairy-sired dairy calves for beef. The Beef + Lamb New Zealand (31) dairy beef integration project demonstrated how “using beef sires with high estimated breeding values for calving ease and growth on dairy farms can provide benefits for both the dairy and beef industries.” A recent Irish initiative, the ICBF's Dairy Beef Index (32), which is a tool that aims to help to promote high quality beef cattle from dairy herds. Preliminary results from the integrated dairy beef project, a collaboration between the ICBF, DAFM, and ICOS (Irish Cooperative Organisation Society) marts, indicate that dairy farmers might be prepared to sacrifice some emphasis on calving/gestation for beef traits (33). Further research and trial programmes will need to be carried out in an Irish context in order to demonstrate to both beef and dairy farmers that they can benefit from greater integration. The messaging surrounding mutual benefits will need to be carefully communicated, given the significant frustration expressed in the qualitative responses by beef, particularly suckler farmers that they are only going to suffer negative consequences by introducing calves of a perceived lower quality into their herds.

### Other Observations

Very few qualitative responses mentioned any of the potential models of dairy beef integration suggested in question sixteen, which may indicate a lack of knowledge among the participants on how these models would operate. One issue which did not arise frequently in the qualitative responses was the potential environmental benefits of greater dairy beef integration. This issue has been examined recently in New Zealand (34) and, given Ireland's obligations to significantly reduce greenhouse gas emissions in the coming years, future research examining the environmental benefits of greater integration in an Irish context would be welcome. Further qualitative research, such as interviews or focus groups, with beef farmers examining the themes arising from this study in greater detail may also provide further insight into these issues. This study has focused almost entirely on a sample of Irish beef farmers. While a large sample of dairy farmers were previously surveyed in respect of calf welfare, it would be useful to examine the attitudes and opinions of this cohort regarding their willingness to facilitate greater integration and their preferred models of doing so, as any potential solution will require unprecedented co-operation between the dairy and beef sectors.

## Conclusions

To the knowledge of the authors, this is one of the first times Irish farmers have been surveyed on dairy beef integration. While many of the qualitative responses were negative in tone, indicating an unwillingness to assist greater integration between beef and dairy enterprise types, a significant cohort of beef farmers already rear dairy calves for beef or are willing to do so in the future. Improvements in respect of the two most frequently occurring themes or perceived barriers from the qualitative analysis, which were price and calf quality, could facilitate greater integration. The following quote was representative of a large number of responses; “*Improve quality of bull calves from dairy herd, fair price from factories for beef it's not rocket science*.” Future strategy will have to consider how these challenges and perceived barriers can be overcome and the issues of who bears the risks and costs associated with greater integration will have to be carefully considered.

## Data Availability Statement

The datasets presented in this article are not readily available because Qualitative free text responses may contain potentially identifiable information. Requests to access the datasets should be directed to annemarie.clarke@agriculture.gov.ie.

## Ethics Statement

Ethical review and approval was not required for the study on human participants in accordance with the local legislation and institutional requirements. The participants provided their written informed consent to participate in this study.

## Author Contributions

MB conceived the idea of the study, reviewed the survey questions, and provided observations on the results. RD suggested questions for the survey and provided observations on the results. AMC and AB contributed to the design of the survey, redrafted the report of the results, and assisted with the literature review. JM assisted with the study design, analysed the quantitative and qualitative data, presented the results to the calf stakeholder forum, and drafted this manuscript. AC assisted with survey design and distribution of the survey. DB oversaw the entire project and contributed to each stage from survey design through to the drafting of this manuscript. All authors read and approved the final manuscript.

## Conflict of Interest

The authors declare that the research was conducted in the absence of any commercial or financial relationships that could be construed as a potential conflict of interest.
