# Supplementary material for: Exploring the Opinions of Irish Beef Farmers Regarding Dairy Beef Integration
Source: Front Vet Sci. 2021 Jun 14;8:660061. doi: 10.3389/fvets.2021.660061 (PMC8238080; doi:10.3389/fvets.2021.660061)
Supplement: Supplementary file 1 [file Data_Sheet_1.PDF]

Q1 Do you consent to participate in this study?

Answered: 1,227 Skipped: 0

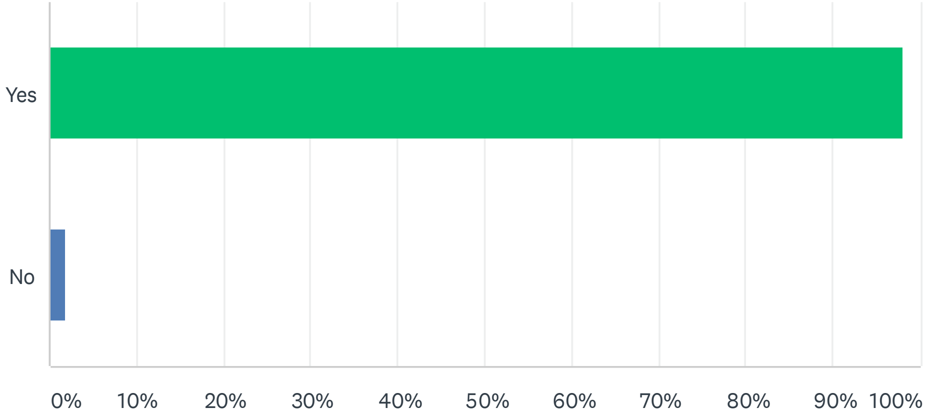

ANSWER CHOICES	RESPONSES	
Yes	98.13%	1,204
No	1.87%	23
TOTAL		1,227

Q2 Age

Answered: 1,162   Skipped: 65

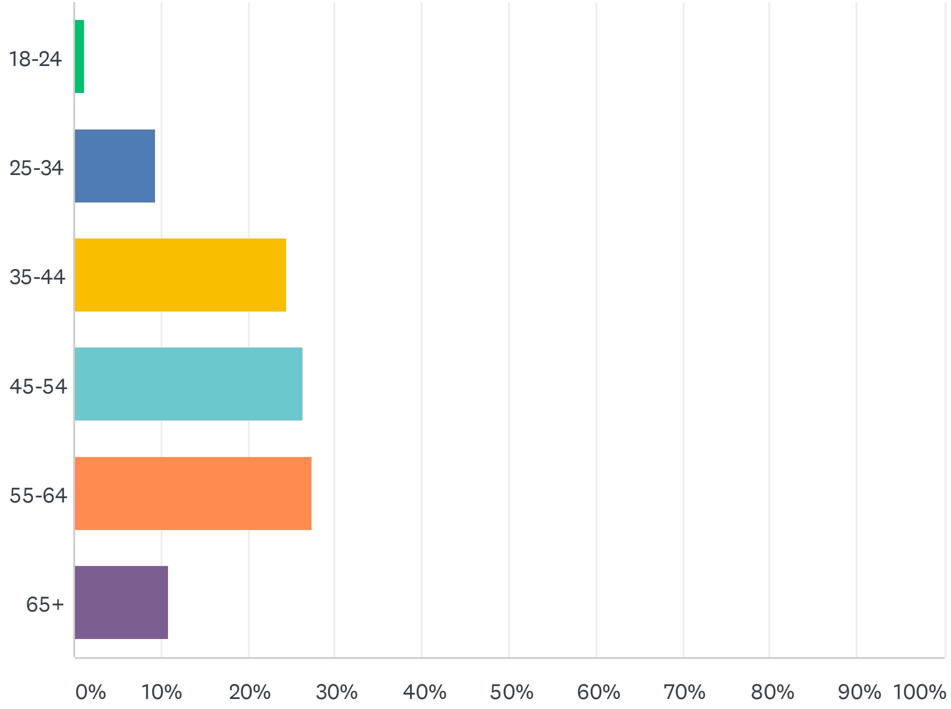

ANSWER CHOICES	RESPONSES	
18-24	1.20%	14
25-34	9.47%	110
35-44	24.53%	285
45-54	26.42%	307
55-64	27.45%	319
65+	10.93%	127
TOTAL		1,162

Q3 Which province do you live in?

Answered: 1,162 Skipped: 65

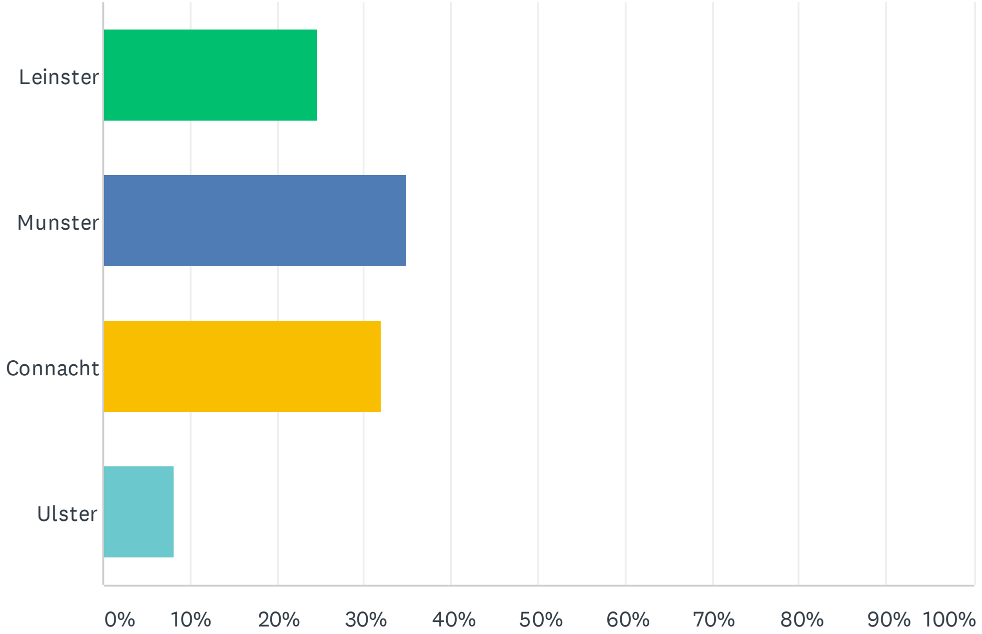

ANSWER CHOICES	RESPONSES	
Leinster	24.78%	288
Munster	35.03%	407
Connacht	31.93%	371
Ulster	8.26%	96
TOTAL		1,162

Q4 Do you farm on a full time or part time basis?

Answered: 1,162 Skipped: 65

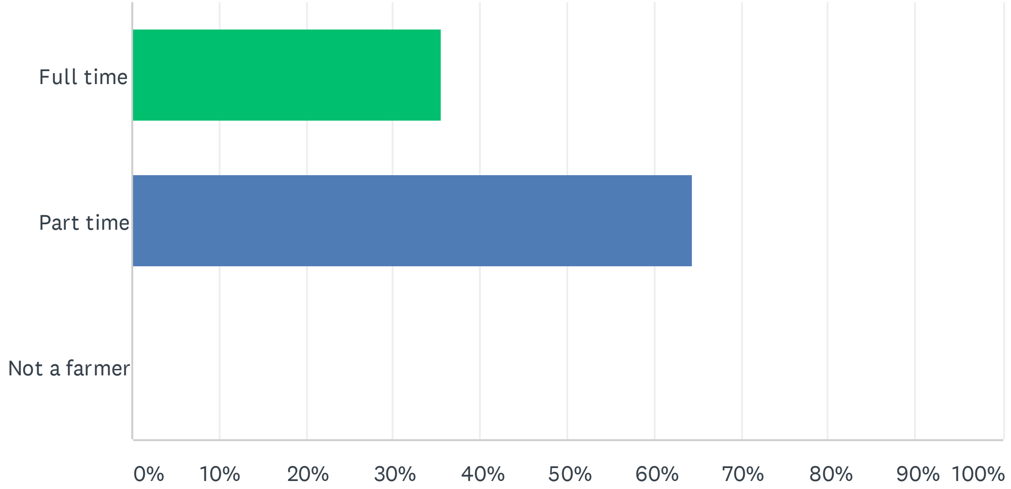

ANSWER CHOICES	RESPONSES	
Full time	35.63%	414
Part time	64.37%	748
Not a farmer	0.00%	0
TOTAL		1,162

Q5 Farm type? Please select all enterprises that apply to you

Answered: 1,162   Skipped: 65

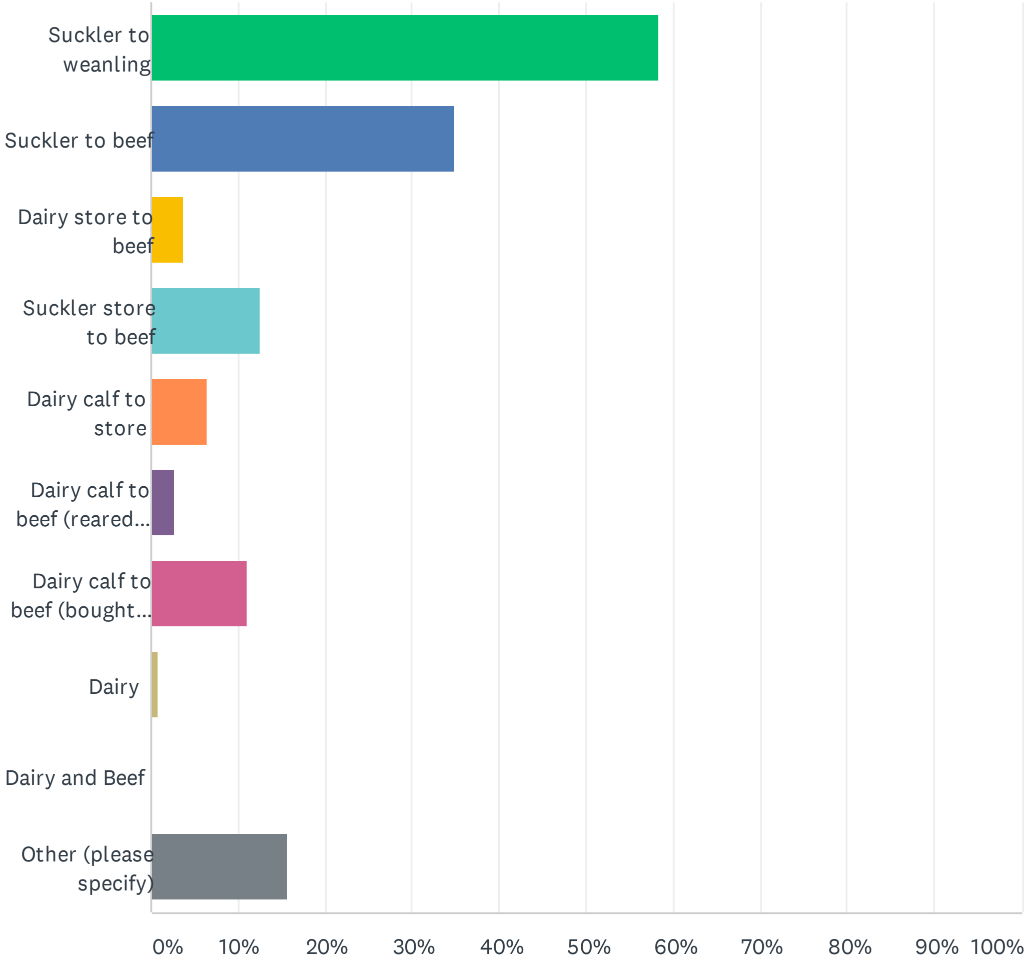

ANSWER CHOICES	RESPONSES	
Suckler to weanling	58.35%	678
Suckler to beef	35.03%	407
Dairy store to beef	3.70%	43
Suckler store to beef	12.56%	146
Dairy calf to store	6.45%	75
Dairy calf to beef (reared on farm)	2.67%	31
Dairy calf to beef (bought in)	11.10%	129
Dairy	0.77%	9
Dairy and Beef	0.00%	0
Other (please specify)	15.75%	183
Total Respondents: 1,162		

Q6 How many cattle do you have on your farm?

Answered: 1,162 Skipped: 65

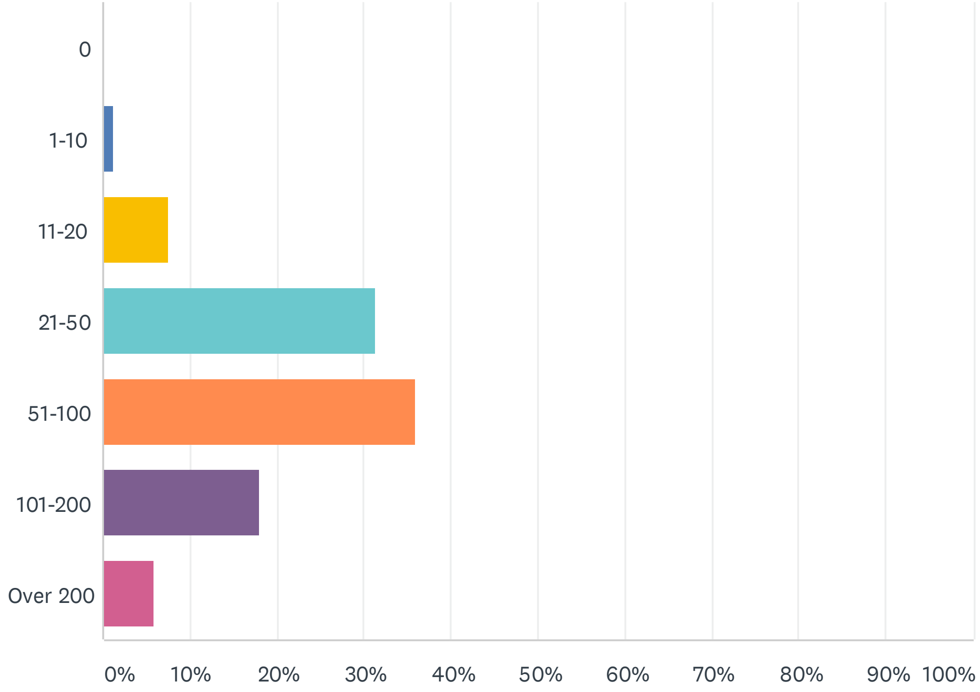

ANSWER CHOICES	RESPONSES	
0	0.09%	1
1-10	1.20%	14
11-20	7.49%	87
21-50	31.41%	365
51-100	35.97%	418
101-200	18.07%	210
Over 200	5.77%	67
TOTAL		1,162

Q7 If you were to rear/finish dairy bred beef, what breed(s) (including cross-bred) would you consider using on your farm? Please select all that apply.

Answered: 1,162 Skipped: 65

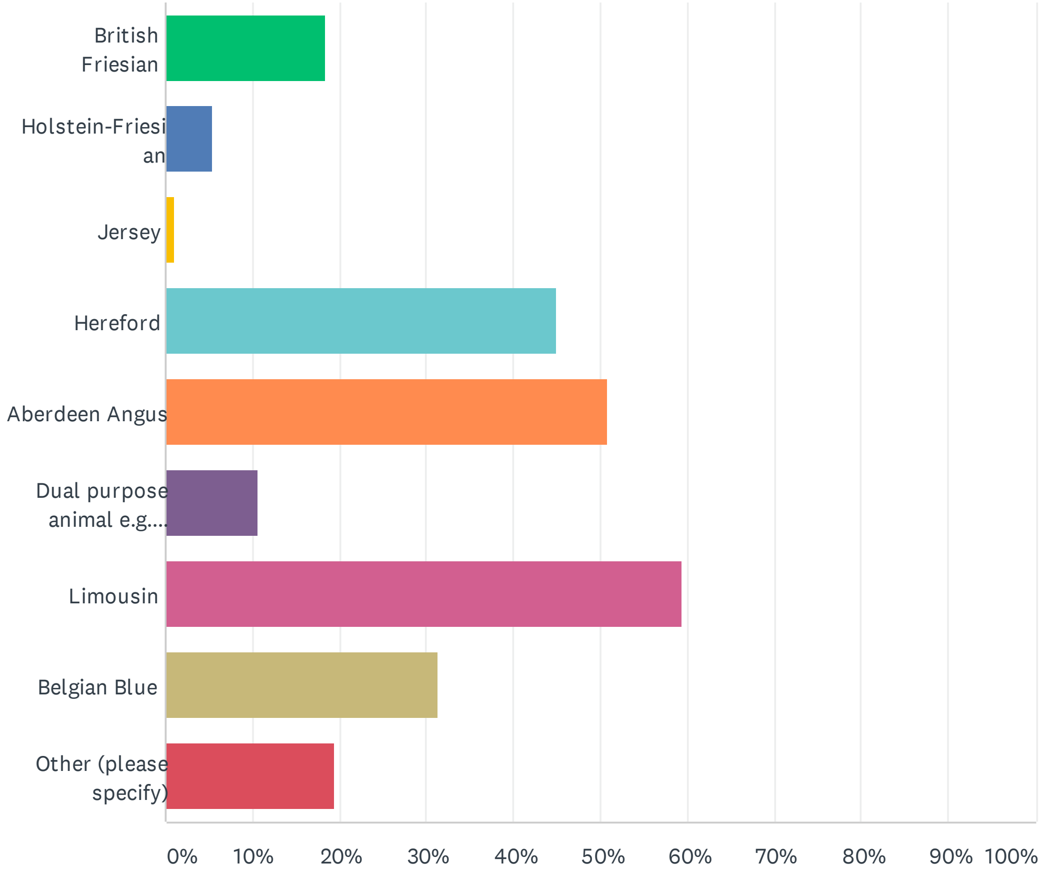

ANSWER CHOICES	RESPONSES	
British Friesian	18.33%	213
Holstein-Friesian	5.34%	62
Jersey	1.12%	13
Hereford	45.01%	523
Aberdeen Angus	50.77%	590
Dual purpose animal e.g. Montbéliarde or Fleckvieh	10.59%	123
Limousin	59.47%	691
Belgian Blue	31.33%	364
Other (please specify)	19.45%	226
Total Respondents: 1,162		

Q8 Are you concerned by the increased number of male dairy calves in recent years?

Answered: 1,041 Skipped: 186

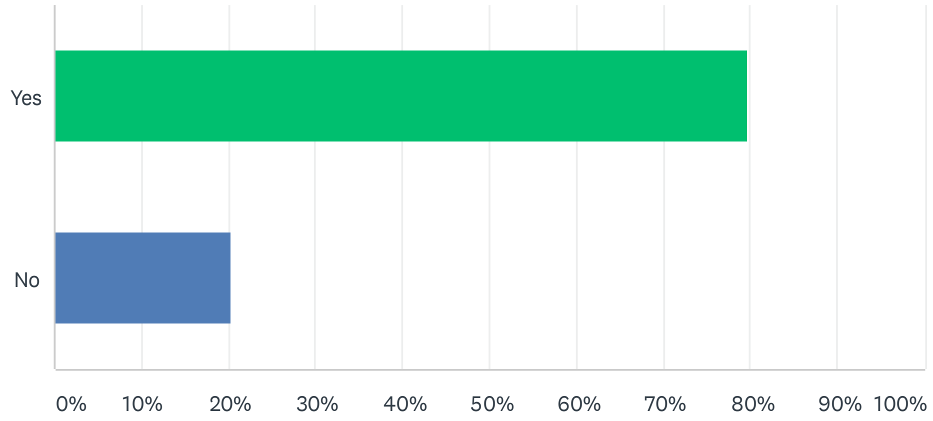

ANSWER CHOICES	RESPONSES	
Yes	79.73%	830
No	20.27%	211
TOTAL		1,041

Q9 Please rank the following factors you think calf rearers would look for when buying animals, with 1 being the most important consideration?

Answered: 1,041 Skipped: 186

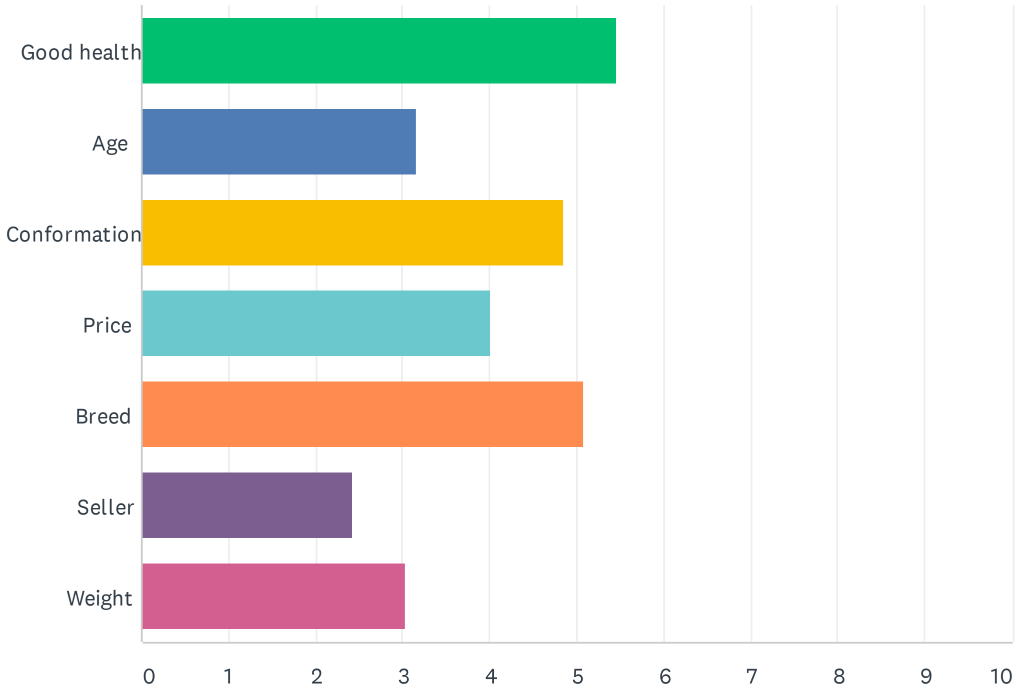

	1	2	3	4	5	6	7	TOTAL	SCORE
Good health	37.66%<br>392	21.52%<br>224	14.99%<br>156	10.76%<br>112	7.11%<br>74	4.90%<br>51	3.07%<br>32	1,041	5.45
Age	3.07%<br>32	6.92%<br>72	11.24%<br>117	17.77%<br>185	20.85%<br>217	22.86%<br>238	17.29%<br>180	1,041	3.16
Conformation	17.68%<br>184	27.38%<br>285	19.50%<br>203	11.91%<br>124	9.70%<br>101	8.65%<br>90	5.19%<br>54	1,041	4.85
Price	10.95%<br>114	12.49%<br>130	16.43%<br>171	19.88%<br>207	17.00%<br>177	14.41%<br>150	8.84%<br>92	1,041	4.02
Breed	24.59%<br>256	22.77%<br>237	20.46%<br>213	14.02%<br>146	8.36%<br>87	5.57%<br>58	4.23%<br>44	1,041	5.08
Seller	3.75%<br>39	4.03%<br>42	6.53%<br>68	9.61%<br>100	13.35%<br>139	17.39%<br>181	45.34%<br>472	1,041	2.42
Weight	2.31%<br>24	4.90%<br>51	10.85%<br>113	16.04%<br>167	23.63%<br>246	26.22%<br>273	16.04%<br>167	1,041	3.03

Q10 Please rank the following factors you think finishers would look for when buying dairy bred beef animals, with 1 being the most important consideration?

Answered: 1,041 Skipped: 186

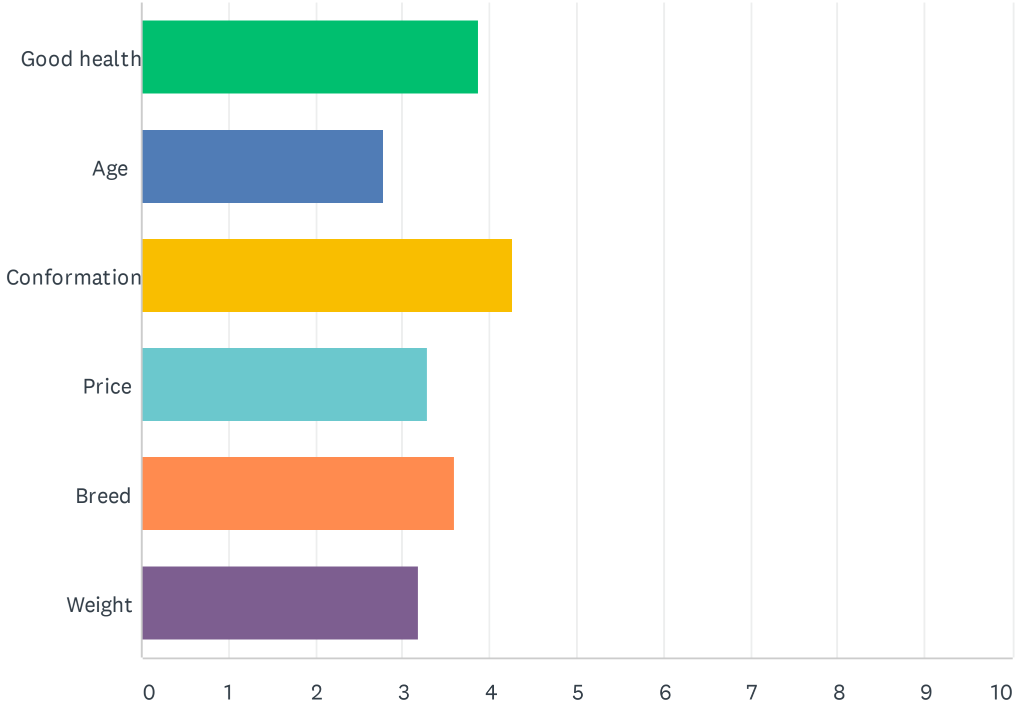

	1	2	3	4	5	6	TOTAL	SCORE
Good health	25.84%<br>269	15.66%<br>163	18.06%<br>188	14.12%<br>147	14.12%<br>147	12.20%<br>127	1,041	3.88
Age	6.05%<br>63	10.28%<br>107	14.41%<br>150	19.88%<br>207	23.44%<br>244	25.94%<br>270	1,041	2.78
Conformation	28.63%<br>298	24.98%<br>260	17.20%<br>179	11.34%<br>118	9.03%<br>94	8.84%<br>92	1,041	4.26
Price	13.83%<br>144	13.26%<br>138	16.14%<br>168	19.79%<br>206	18.06%<br>188	18.92%<br>197	1,041	3.28
Breed	17.48%<br>182	19.60%<br>204	16.23%<br>169	15.37%<br>160	14.60%<br>152	16.71%<br>174	1,041	3.60
Weight	8.17%<br>85	16.23%<br>169	17.96%<br>187	19.50%<br>203	20.75%<br>216	17.39%<br>181	1,041	3.19

Q11 Are you willing to incorporate the rearing of dairy bred calves for beef into your farm enterprise? Please select all options that apply to you.

Answered: 1,041   Skipped: 186

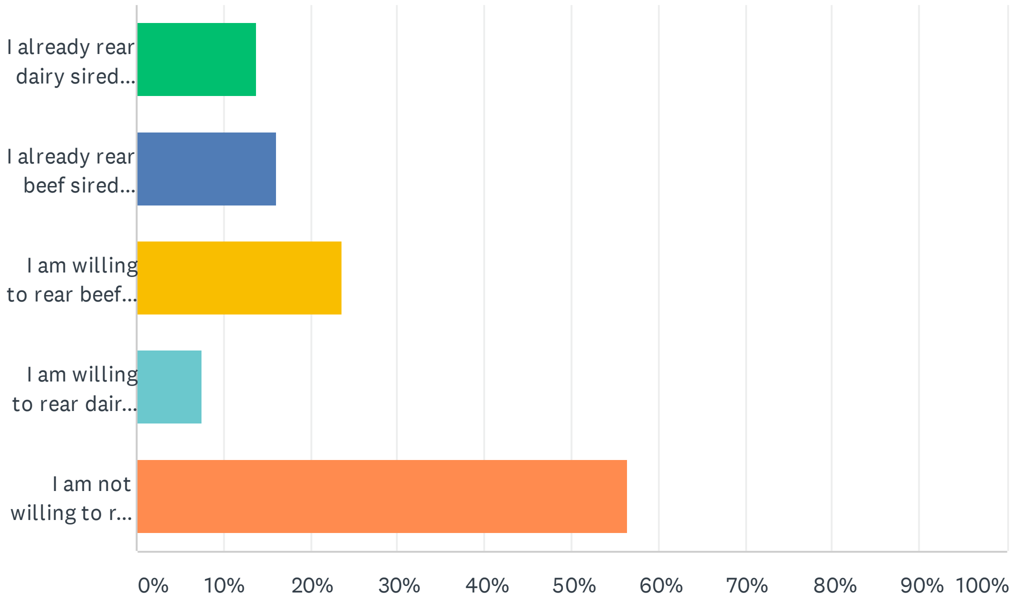

ANSWER CHOICES	RESPONSES	
I already rear dairy sired calves for beef	13.83%	144
I already rear beef sired dairy calves for beef	16.14%	168
I am willing to rear beef sired dairy calves for beef	23.63%	246
I am willing to rear dairy sired calves for beef	7.49%	78
I am not willing to rear any dairy bred calves for beef	56.58%	589
Total Respondents: 1,041		

Q12 How important to you is genomic verification (for breed and genetic merit) when buying calves?

Answered: 1,038   Skipped: 189

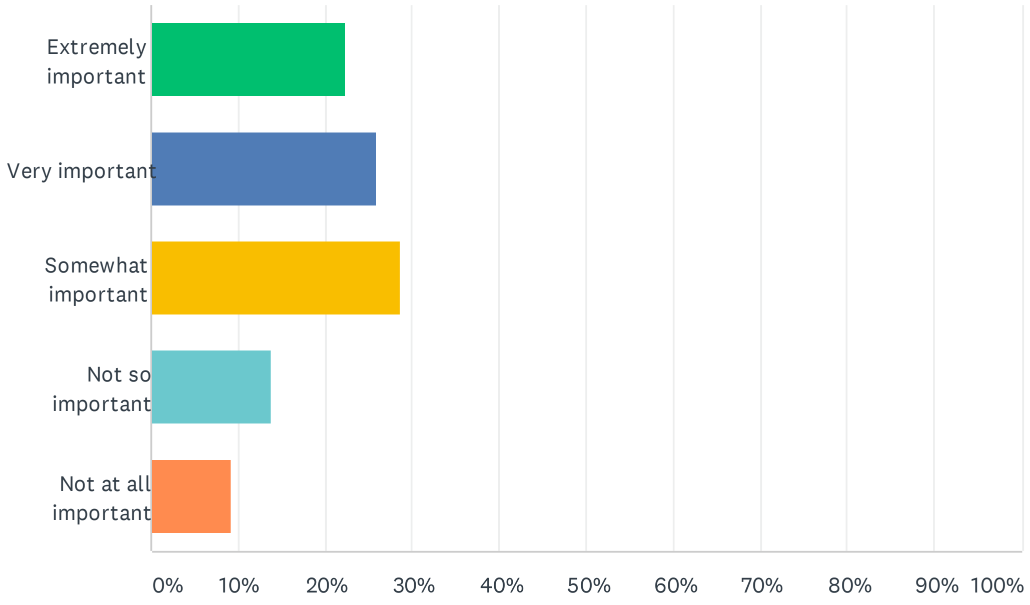

ANSWER CHOICES	RESPONSES	
Extremely important	22.35%	232
Very important	25.92%	269
Somewhat important	28.61%	297
Not so important	13.87%	144
Not at all important	9.25%	96
TOTAL		1,038

Q13 Please rank the following factors that would dissuade you from rearing dairy calves for beef, with 1 being the most important factor?

Answered: 918 Skipped: 309

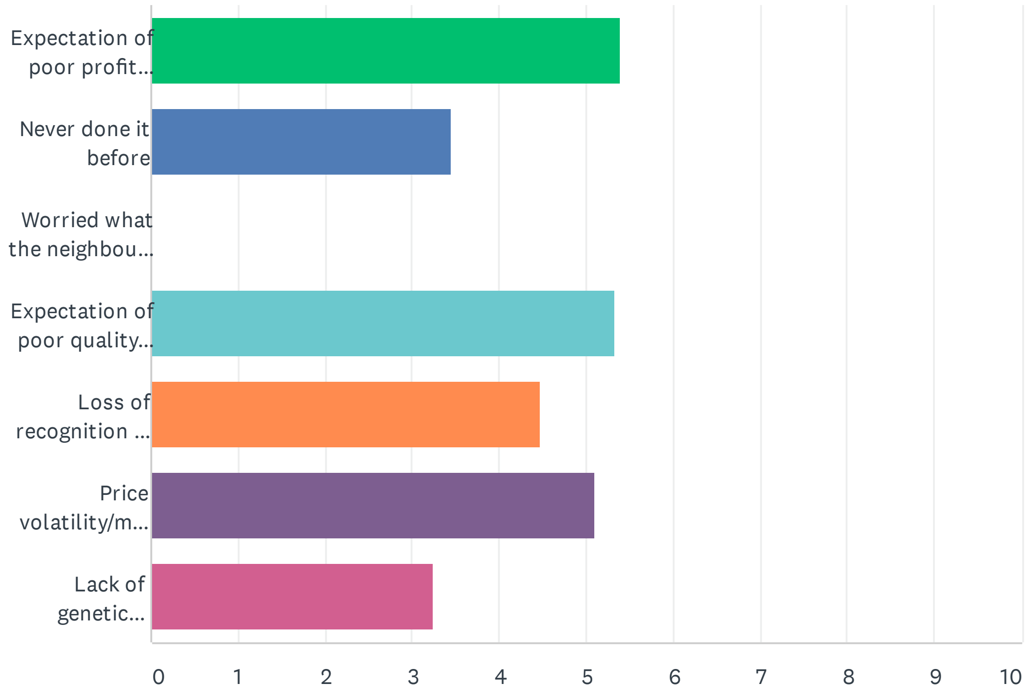

	1	2	3	4	5	6	7	TOTAL	SCORE
Expectation of poor profit margin	25.82%<br>237	27.23%<br>250	21.24%<br>195	14.49%<br>133	8.39%<br>77	2.83%<br>26	0.00%<br>0	918	5.39
Never done it before	11.44%<br>105	5.56%<br>51	7.73%<br>71	11.55%<br>106	19.93%<br>183	43.79%<br>402	0.00%<br>0	918	3.46
Worried what the neighbours would think	0.00%<br>0	0.00%<br>0	0.00%<br>0	0.00%<br>0	0.00%<br>0	0.00%<br>0	0.00%<br>0	0	0.00
Expectation of poor quality animals	23.75%<br>218	26.36%<br>242	23.09%<br>212	16.56%<br>152	7.73%<br>71	2.51%<br>23	0.00%<br>0	918	5.34
Loss of recognition as quality beef producer	16.78%<br>154	12.20%<br>112	15.47%<br>142	22.44%<br>206	22.98%<br>211	10.13%<br>93	0.00%<br>0	918	4.47
Price volatility/market uncertainty	19.39%<br>178	22.77%<br>209	23.64%<br>217	19.93%<br>183	11.00%<br>101	3.27%<br>30	0.00%<br>0	918	5.10
Lack of genetic information available e.g. sire confirmation, breed breakdown	2.83%<br>26	5.88%<br>54	8.82%<br>81	15.03%<br>138	29.96%<br>275	37.47%<br>344	0.00%<br>0	918	3.24

Q14 Please rank the following barriers to you rearing dairy bred calves, with one being the most significant barrier?

Answered: 918 Skipped: 309

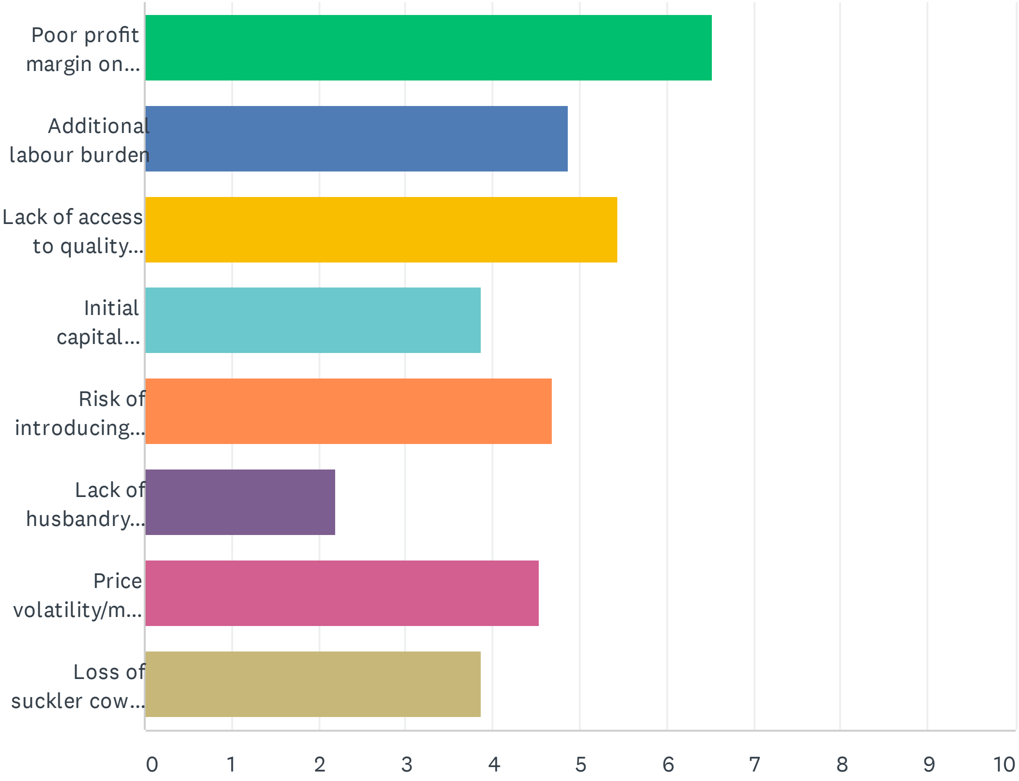

	1	2	3	4	5	6	7	8	TOTAL	SCORE
Poor profit margin on investment	41.50% 381	21.02% 193	15.03% 138	8.17% 75	6.43% 59	3.27% 30	2.40% 22	2.18% 20	918	6.53
Additional labour burden	9.26% 85	17.76% 163	15.80% 145	16.67% 153	13.62% 125	10.57% 97	9.48% 87	6.86% 63	918	4.88
Lack of access to quality calves	13.62% 125	18.52% 170	22.66% 208	15.58% 143	12.64% 116	9.48% 87	5.56% 51	1.96% 18	918	5.44
Initial capital investment required	2.61% 24	5.88% 54	10.24% 94	16.23% 149	18.95% 174	22.00% 202	15.69% 144	8.39% 77	918	3.86
Risk of introducing disease	10.24% 94	10.24% 94	13.73% 126	16.01% 147	20.04% 184	15.69% 144	10.68% 98	3.38% 31	918	4.68
Lack of husbandry skills	0.54% 5	0.87% 8	1.63% 15	4.14% 38	6.43% 59	19.06% 175	27.45% 252	39.87% 366	918	2.19
Price volatility/market uncertainty	8.93% 82	16.01% 147	12.09% 111	14.38% 132	12.31% 113	11.76% 108	16.34% 150	8.17% 75	918	4.53
Loss of suckler cow assets	13.29% 122	9.69% 89	8.82% 81	8.82% 81	9.59% 88	8.17% 75	12.42% 114	29.19% 268	918	3.88

Q15 Please rank the following potential strategies in order of how effective you think they would be in improving dairy beef integration, with 1 being most effective.

Answered: 918 Skipped: 309

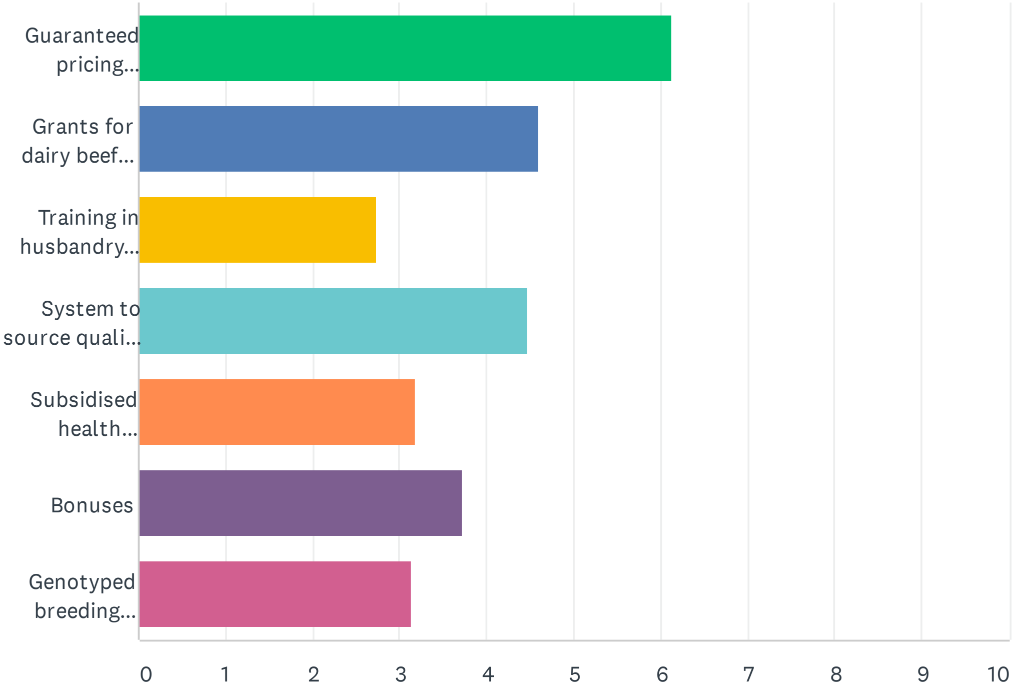

	1	2	3	4	5	6	7	TOTAL	SCORE
Guaranteed pricing mechanisms	58.28% 535	18.74% 172	11.00% 101	5.45% 50	3.16% 29	1.96% 18	1.42% 13	918	6.12
Grants for dairy beef infrastructure (e.g. weight recording equipment, automatic feeders, calf handling facilities)	12.85% 118	28.43% 261	17.97% 165	12.20% 112	11.66% 107	10.13% 93	6.75% 62	918	4.61
Training in husbandry skills	1.31% 12	6.54% 60	12.75% 117	12.31% 113	13.62% 125	19.83% 182	33.66% 309	918	2.75
System to source quality calves	11.44% 105	17.10% 157	20.59% 189	25.16% 231	12.42% 114	10.02% 92	3.27% 30	918	4.47
Subsidised health programmes	0.98% 9	4.47% 41	10.46% 96	19.06% 175	35.84% 329	20.15% 185	9.04% 83	918	3.19
Bonuses	4.47% 41	15.47% 142	19.06% 175	14.92% 137	12.20% 112	23.09% 212	10.78% 99	918	3.73
Genotyped breeding programme to produce better calves	10.68% 98	9.26% 85	8.17% 75	10.89% 100	11.11% 102	14.81% 136	35.08% 322	918	3.13

Q16 Please rank the following potential models of dairy beef integration in order of preference, with 1 being your favourite model.

Answered: 902 Skipped: 325

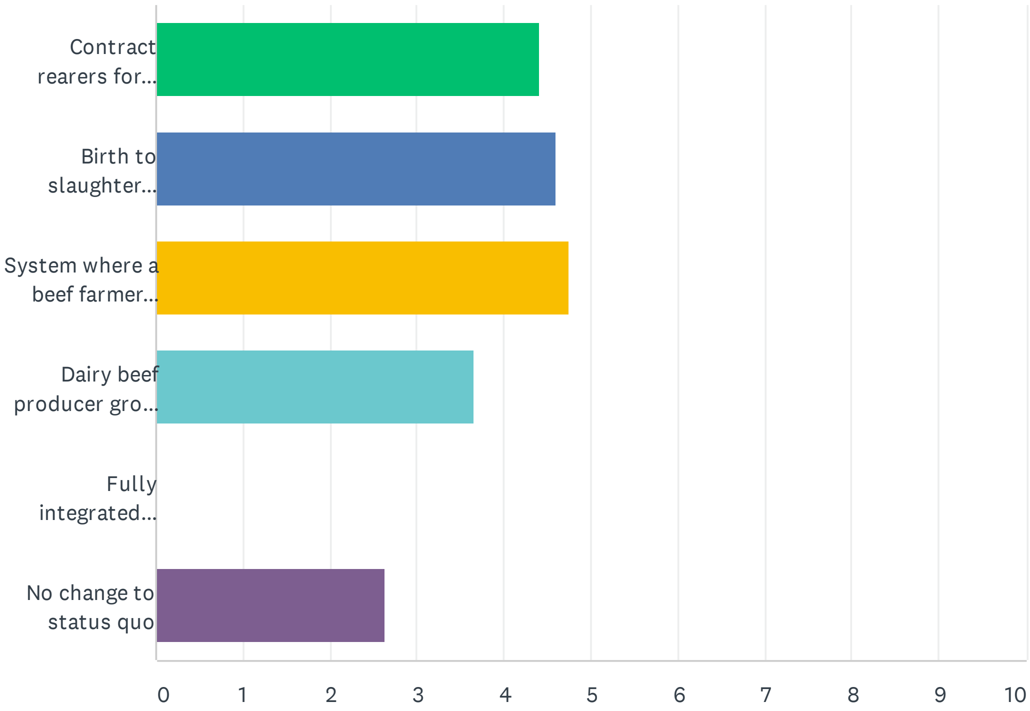

	1	2	3	4	5	6	TOTAL	SCORE
Contract rearers for dairy farmers (Beef farmers are paid by the dairy farmer to finish the dairy bred animals)	28.98% 255	21.70% 191	21.82% 192	16.36% 144	11.14% 98	0.00% 0	880	4.41
Birth to slaughter production contract system (Fully integrated system where there are contracts between the dairy farmer to supply the beef farmer with an agreed type of calf, and a contract with the beef processor to supply minimum agreed price (or bonus system) to the beef farmer who rears this animal to the agreed specification)	26.41% 234	31.60% 280	23.81% 211	12.42% 110	5.76% 51	0.00% 0	886	4.60
System where a beef farmer rears and/or finishes dairy bred calves with guaranteed pricing mechanism, with the beef farmer retaining ownership of the calves (e.g. Glanbia Kepak beef club)	32.77% 292	28.96% 258	23.23% 207	11.00% 98	4.04% 36	0.00% 0	891	4.75
Dairy beef producer group e.g. Specific breed producer group	6.19% 55	14.64% 130	24.66% 219	47.75% 424	6.76% 60	0.00% 0	888	3.66
Fully integrated system e.g. Blade	0.00% 0	0.00% 0	0.00% 0	0.00% 0	0.00% 0	0.00% 0	0	0.00
No change to status quo	7.17% 64	3.47% 31	5.94% 53	12.32% 110	71.11% 635	0.00% 0	893	2.63

Q17 Please provide any suggestions you have for how dairy beef animals could be better integrated into beef rearing/finishing systems?

Answered: 541 Skipped: 686
