# Supplementary material for: Exploring the Opinions of Irish Beef Farmers Regarding Dairy Beef Integration
Source: Front Vet Sci. 2021 Jun 14;8:660061. doi: 10.3389/fvets.2021.660061 (PMC8238080; doi:10.3389/fvets.2021.660061)
Supplement: Supplementary file 2 [file Table_1.DOCX]

**Additional file 1: Survey questions**

1. *Do you consent to participate in this study?* Yes; No.
2. *Age?* 18-24; 25-34; 35-44; 45-54; 55-64; 65+.
3. *Which province do you live in?* Leinster; Munster; Connacht; Ulster.
4. *Do you farm on a full time or part time basis?* Full time; Part time; Not a farmer.
5. *Farm type? Please select all enterprises that apply to you*. Suckler to weanling; Suckler to beef; Dairy store to beef; Suckler store to beef; Dairy calf to store; Dairy calf to beef (reared on farm); Dairy calf to beef (bought in); Dairy; Other (please specify).
6. *How many cattle do you have on your farm?* 0; 1-10; 11-20; 21-50; 51-100; 101-200; Over 200.
7. *If you were to rear/finish dairy bred beef, what breed(s) (including cross-bred) would you consider using on your farm? Please select all that apply*. Jersey; Aberdeen Angus; Limousin; Dual purpose animal e.g. Montbéliarde or Fleckvieh; Holstein-Friesian; Hereford; British Friesian; Belgian Blue; Other (please specify).
8. *Are you concerned by the increased number of male dairy calves in recent years?* Yes; No.
9. *Please rank the following factors you think calf rearers would look for when buying animals, with 1 being the most important consideration.* Age; Seller; Weight; Conformation; Breed; Good health; Price.
10. *Please rank the following factors you think finishers would look for when buying dairy bred beef animals, with 1 being the most important consideration.* Age; Weight; Conformation; Breed; Good health; Price.
11. *Are you willing to incorporate the rearing of dairy bred calves for beef into your farm enterprise?* Please select all options that apply to you. I already rear dairy sired calves for beef; I already rear beef sired dairy calves for beef; I am willing to rear beef sired dairy calves for beef; I am willing to rear dairy sired calves for beef; I am not willing to rear any dairy bred calves for beef.
12. *How important to you is genomic verification (for breed and genetic merit) when buying calves?* Extremely important; Very important; Somewhat important; Not so important; Not at all important.
13. *Please rank the following factors that would dissuade you from rearing dairy calves for beef, with 1 being the most important factor.* Price volatility/market uncertainty; Expectation of poor quality animals; Never done it before; Loss of recognition as quality beef producer; Expectation of poor product margin; Lack of genetic information available e.g. sire confirmation, breed breakdown.
14. *Please rank the following barriers to you rearing dairy bred calves, with one being the most significant barrier*. Poor profit margin on investment; Additional labour burden; Lack of access to quality calves; Initial capital investment required; Risk of introducing disease; Lack of husbandry skills; Price volatility/market uncertainty; Loss of suckler cow assets.
15. *Please rank the following potential strategies in order of how effective you think they would be in improving dairy beef integration, with 1 being most effective*. Guaranteed pricing mechanisms; Grants for dairy beef infrastructure (e.g. weight recording equipment, automatic feeders, calf handling facilities); Training in husbandry skills; System to source quality calves; Subsidised health programmes; Bonuses; Genotyped breeding programme to produce better calves.
16. *Please rank the following potential models of dairy beef integration in order of preference, with 1 being your favourite model.* Contract rearers for dairy farmers (Beef farmers are paid by the dairy farmer to finish the dairy bred animals); Birth to slaughter production contract system (Fully integrated system where there are contracts between the dairy farmer to supply the beef farmer with an agreed type of calf, and a contract with the beef processor to supply minimum agreed price (or bonus system) to the beef farmer who rears this animal to the agreed specification); System where a beef farmer rears and/or finishes dairy bred calves with guaranteed pricing mechanism, with the beef farmer retaining ownership of the calves (e.g. Glanbia Kepak beef club); Dairy beef producer group e.g. Specific breed producer group; Fully integrated system e.g. Blade; No change to status quo.
17. *Please provide any suggestions you have for how dairy beef animals could be better integrated into beef rearing/finishing systems.*
